# Supplementary material for: GABAA receptor dependent synaptic inhibition rapidly tunes KCC2 activity via the Cl−-sensitive WNK1 kinase
Source: Nat Commun. 2017 Nov 24;8:1776. doi: 10.1038/s41467-017-01749-0 (PMC5701213; doi:10.1038/s41467-017-01749-0)
Supplement: Supplementary file 1 — Supplementary Information [file 41467_2017_1749_MOESM1_ESM.pdf]

Supplementary Figures

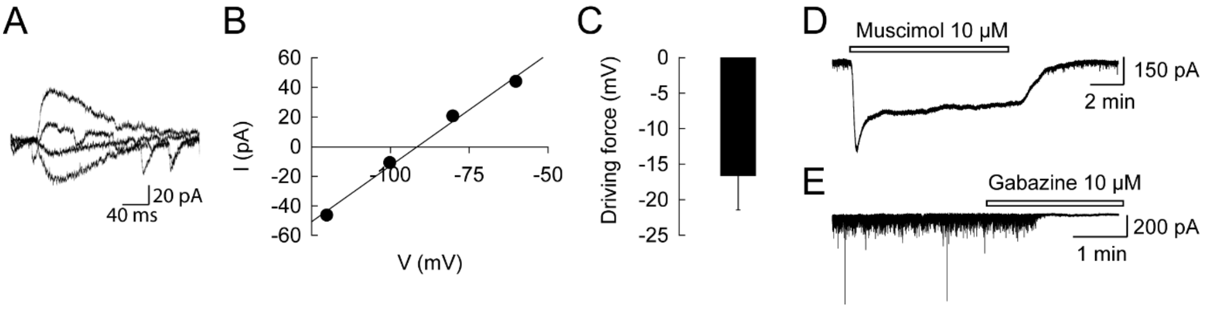

Supplementary figure 1. E_{GABA} and effects of muscimol and gabazine. A-B, *Typical gramicidin-perforated patch-clamp recordings of GABAA receptor mediated currents induced by uncaging of Rubi-GABA at different membrane potentials. E_{GABA} was determined as the intercept of the I-V curve with the x-axis.* ***C****, The driving force for chloride (Cl^-) is hyperpolarizing indicating chloride influx upon $GABA_AR$ activation. N= 11 cells.* ***D****, Whole-cell patch-clamp recording of a hippocampal neuron with a CsMeSO4-based internal solution before, during and after application of the GABAAR agonist muscimol. Muscimol-induced current peaked within approximately 30 s of wash-in and decayed by about 50 % upon 10 minutes of agonist application.* ***E,*** *Whole-cell patch-clamp recording of a hippocampal neuron with a CsCl-based internal solution, before and during application of the GABAAR antagonist gabazine, in the presence of TTX, NBQX and D,L-APV. Gabazine efficiently suppressed mIPSCs with no detectable change in holding current, suggesting it did not affect tonic, GABAAR-mediated currents.*

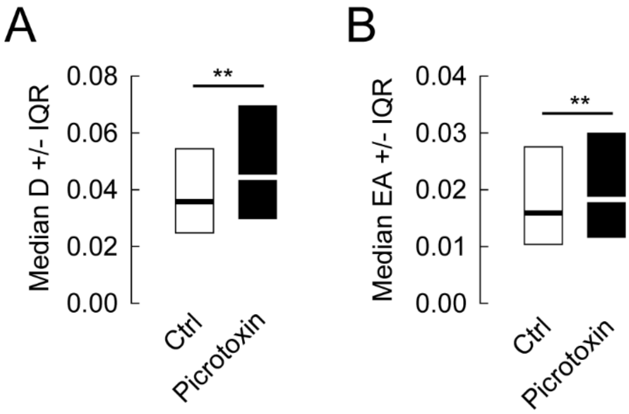

Supplementary figure 2. The pore channel blocker picrotoxin relieves KCC2 diffusion constraints. A-B, *Median diffusion coefficients D values ± 25-75% IQR (**A**) and median explored area EA ± 25%-75% IQR (**B**) (for bulk population of QDs) of KCC2 measured in control (white) vs picrotoxin (black) conditions showing picrotoxin reduces KCC2 diffusion constraints.* ***A****, n= 227 QDs, 2 cultures; KS test p=0.002.* ***B****, n= 554 QDs, 2 cultures; KS test p=0.009.* ***A****, D in $\mu m^2 s^{-1}$;* ***B****, EA in μm^2.*

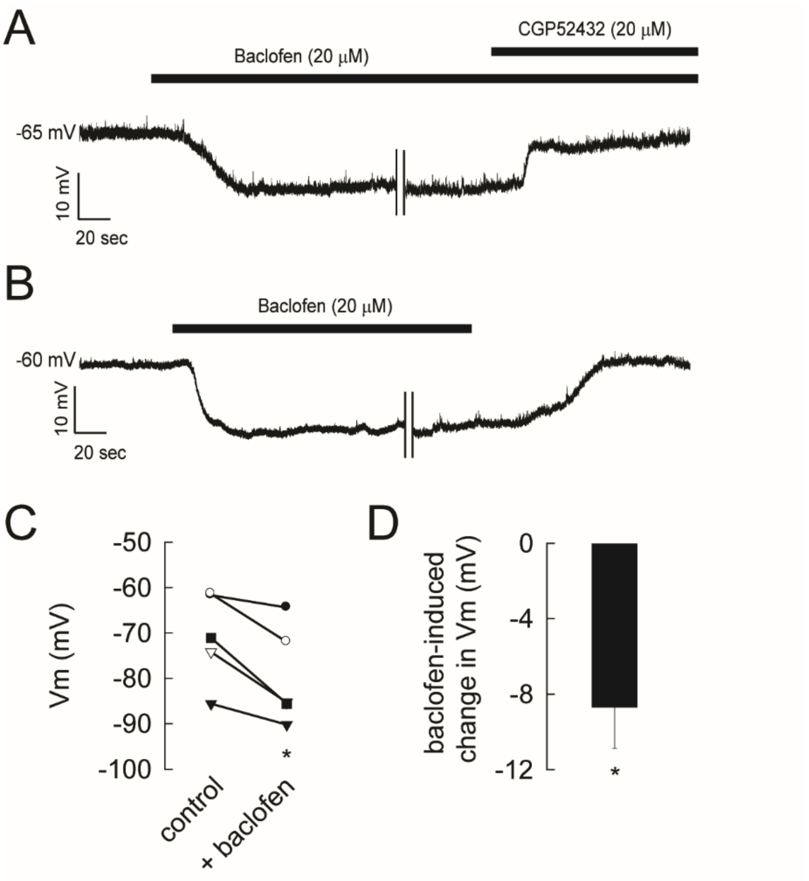

Supplementary figure 3. Activation of $GABA_BR$ hyperpolarizes V_m in cultured hippocampal neurons. A-B, *Representative trace of V_m measured by whole-cell patch clamp recordings in cultured hippocampal neurons (DIV 21-24) bathed in TTX (1 µM), kynurenate (1 mM), and MCPG (500 µM) before and during application of the $GABA_BR$ agonist baclofen (20 µM). Vertical lines depict a break in recording time followed by blocking $GABA_BR$ activity using CGP52432 (20 µM, **A**) or during washout of baclofen (**B**). V_m returns to baseline in response to the addition of CGP52432 and after washing out baclofen. **C**, Scatter plot of V_m measurements before and during the application of baclofen. N= 5 cells; paired t-test p =0.016. **D,** Bar graph summarizing the change in V_m observed during the application of baclofen. Bar represents mean change in V_m ± s.e.m. N= 5 cells, paired t-test, p =0.016.*

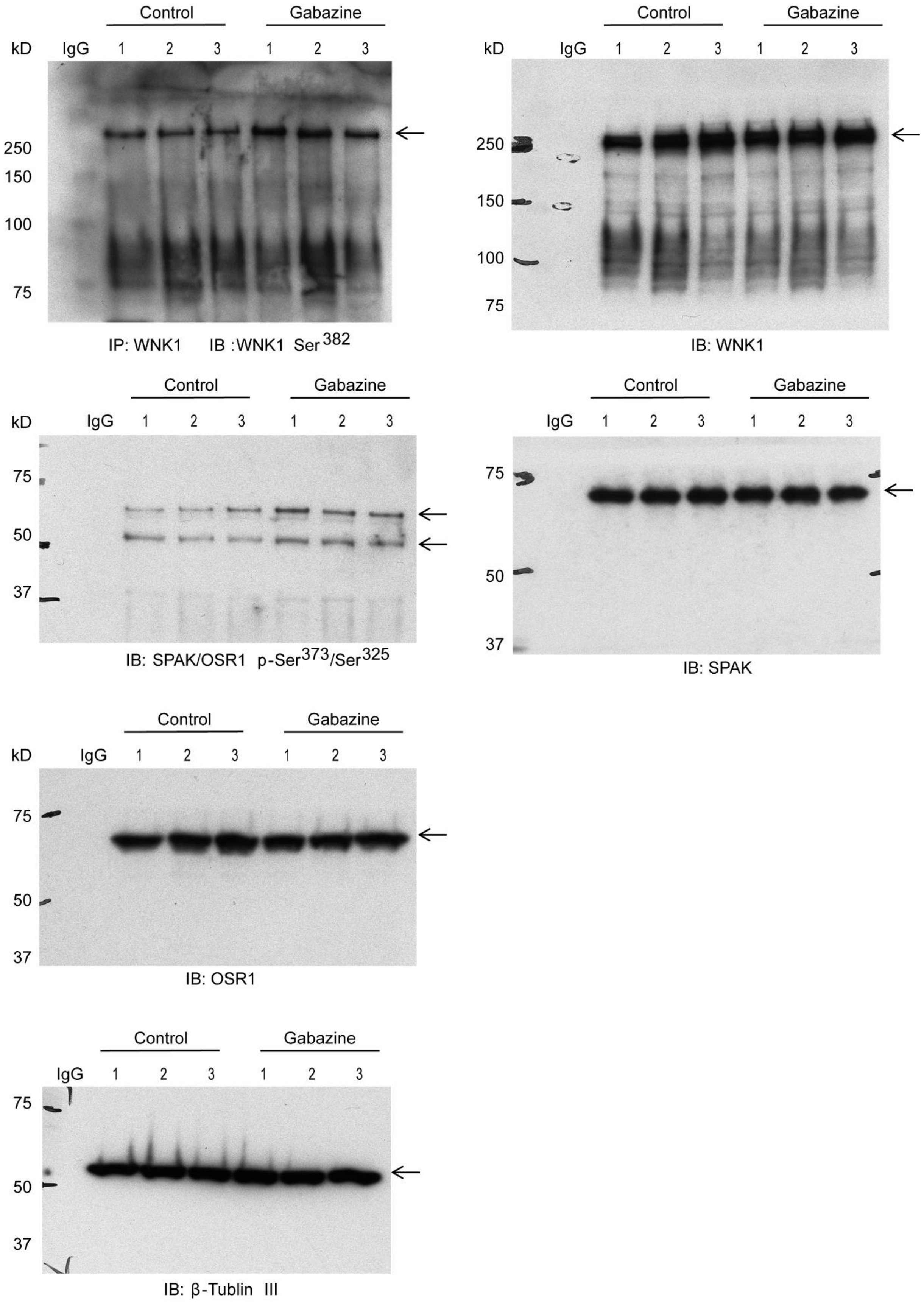

Supplementary Figure 4. Full size immunoblots related to Fig. 5C. *Molecular weight markers positions (kD).*

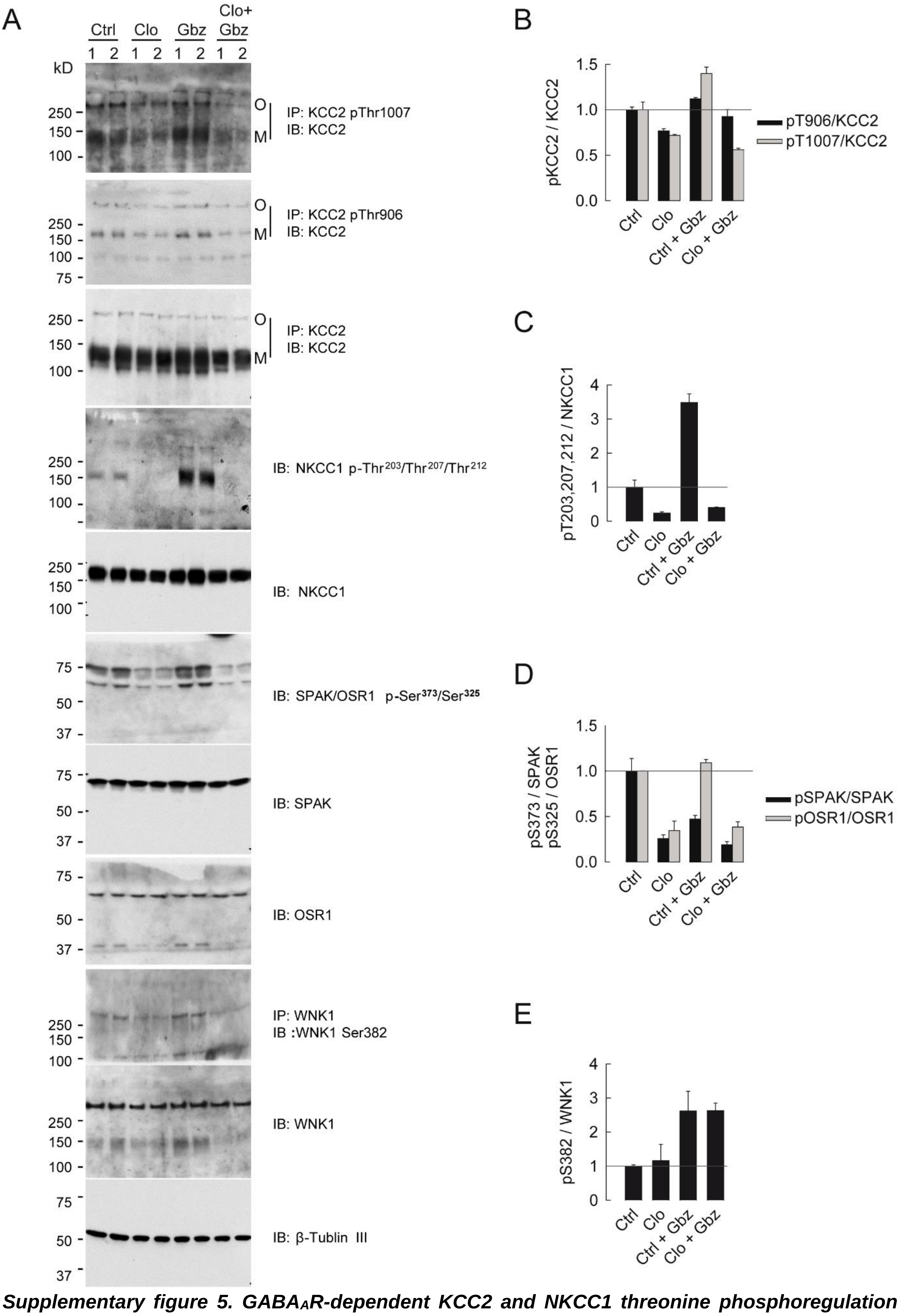

***Supplementary figure 5. GABA$_A$R-dependent KCC2 and NKCC1 threonine phosphoregulation require SPAK/OSR1 kinase activity. A-E**, Western Blot (**A**) and quantification (mean ± s.e.m., two independent experiments 1-2) of KCC2 T906/T1007 (**B**), NKCC1 T203/T207/T212 (**C**), SPAK S373 / OSR1 S325 (**D**) and WNK1 S382 (**E**) kinases phosphorylation in control (Ctrl), gabazine (Ctrl+Gbz),*

closantel (Clo) and closantel+gabazine (Clo+Gbz) conditions. Note closantel inactivates SPAK/OSR kinases but not the upstream kinase WNK1 and blocks the increased phosphorylation of KCC2 and NKCC1 upon GABA$_A$R blockade. O, oligomers; M, monomers.

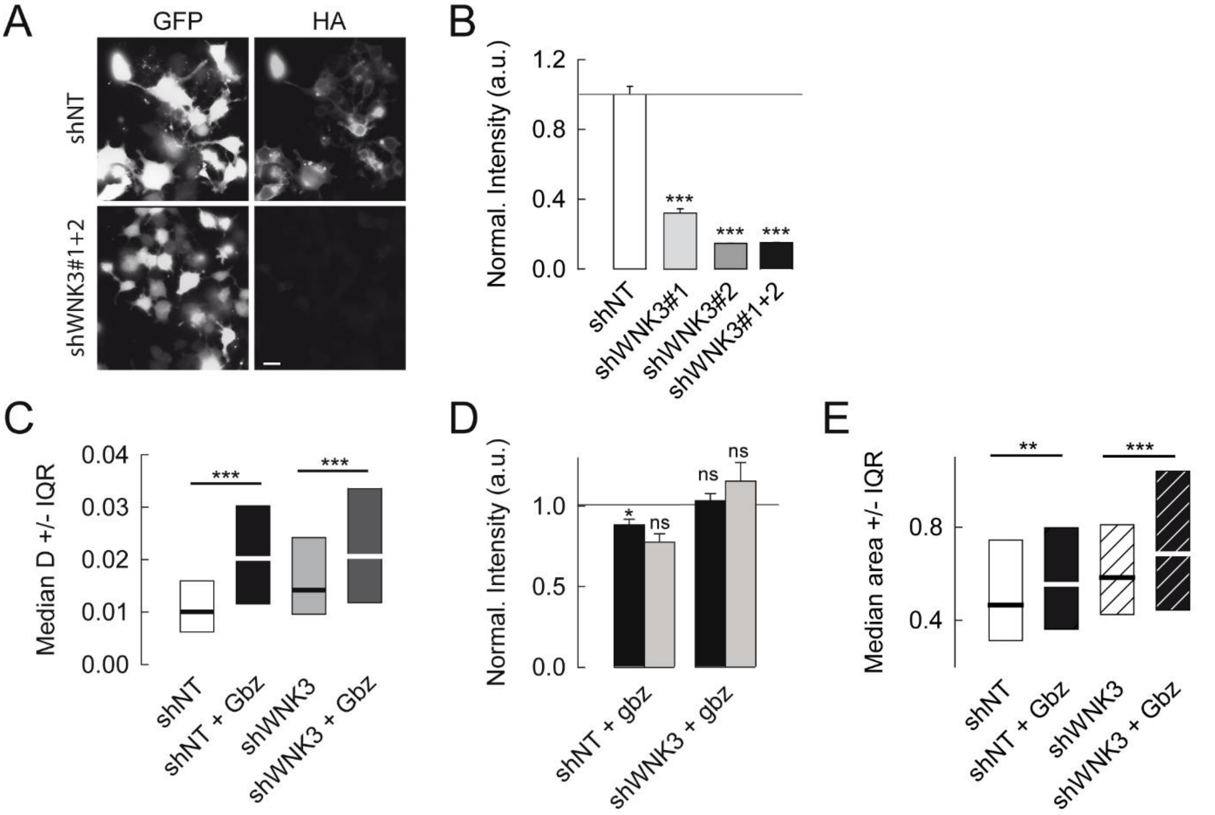

***Supplementary figure 6. WNK3 suppression partially blocks gabazine-induced regulation of KCC2. A-B**, Characterization of rat WNK3 shRNAs. **A**, HA immunostaining of Neuro2A cells co-transfected with a recombinant HA-tagged rat WNK3 cDNA and either a GFP-tagged non-target shRNA (shNT) or a cocktail of two GFP-tagged shRNAs against WNK3 (shWNK3#1+2). Scale bar, 20 µm. **B**, Quantification of the average fluorescence intensity of HA-labeled WNK3 in Neuro2A cells expressing shNT, WNK3 shRNA #1 (shWNK3#1), #2 (shWNK3#2) or a cocktail of #1+2 (shWNK3#1+2). Values were normalized to the shNT mean fluorescence intensity value. The efficiency of shWNK3#1, shWNK3#2 or shWNK3#1+2 is respectively of 68%, 79% and 83%. shNT n= 582 cells, shWNK3#1 n= 539 cells, shWNK3#2 n= 774 cells, shWNK3#1+2 n= 681 cells; MW test p<0.001. **C**, WNK3 suppression by shRNA (shWNK3) did not abolish the gabazine-mediated increase in KCC2 diffusion. shNT n= 322 QDs, shNT + Gbz 322 QDs, shWNK3 330 QDs, shWNK3 + Gbz 330 QDs, 2 cultures, shNT vs shNT + Gbz KS test p<0.001, shWNK3 vs shWNK3 + Gbz KS test p<0.001. **D**, WNK3 shRNA (shWNK3) overexpression suppressed the gabazine-induced reduction in cluster (gray) and pixel (black) fluorescence intensity as compared to shNT expressing neurons. Values were normalized to the corresponding control values. shNT: Ctrl 70 cells, Gbz n= 73 cells; shWNK3: Ctrl n= 77 cells, Gbz n= 71 cells; 4-5 cultures. shNT vs shNT + Gbz: MW test p=0.018 and p= 0.114 for pixel and cluster intensity respectively; shWNK3 vs shNT: MW test p=0.004 andp=0.004 for pixel and cluster intensity; shWNK3 vs shWNK3 + Gbz: MW test p=0.954 and p=0.797 for pixel and cluster intensity. **E,** Median values ± 25-75% IQR of spine head area from shNT or shWNK3 overexpressing neurons in control (white and black stripe) or gabazine (black and white stripe) conditions, respectively. Note the increase in spine head area in gabazine condition for shNT (KS test p=0.0011) and shWNK3 (KS test p<0.001) transfected cells. ShNT n= 486 spines; shNT + Gbz n= 244 spines; shWNK3 n= 266 spines, shWNK3 + Gbz n= 291 spines, 2 cultures. **C,** D in µm^{2}s^{-1}; **E,** spine head area in µm^2.*

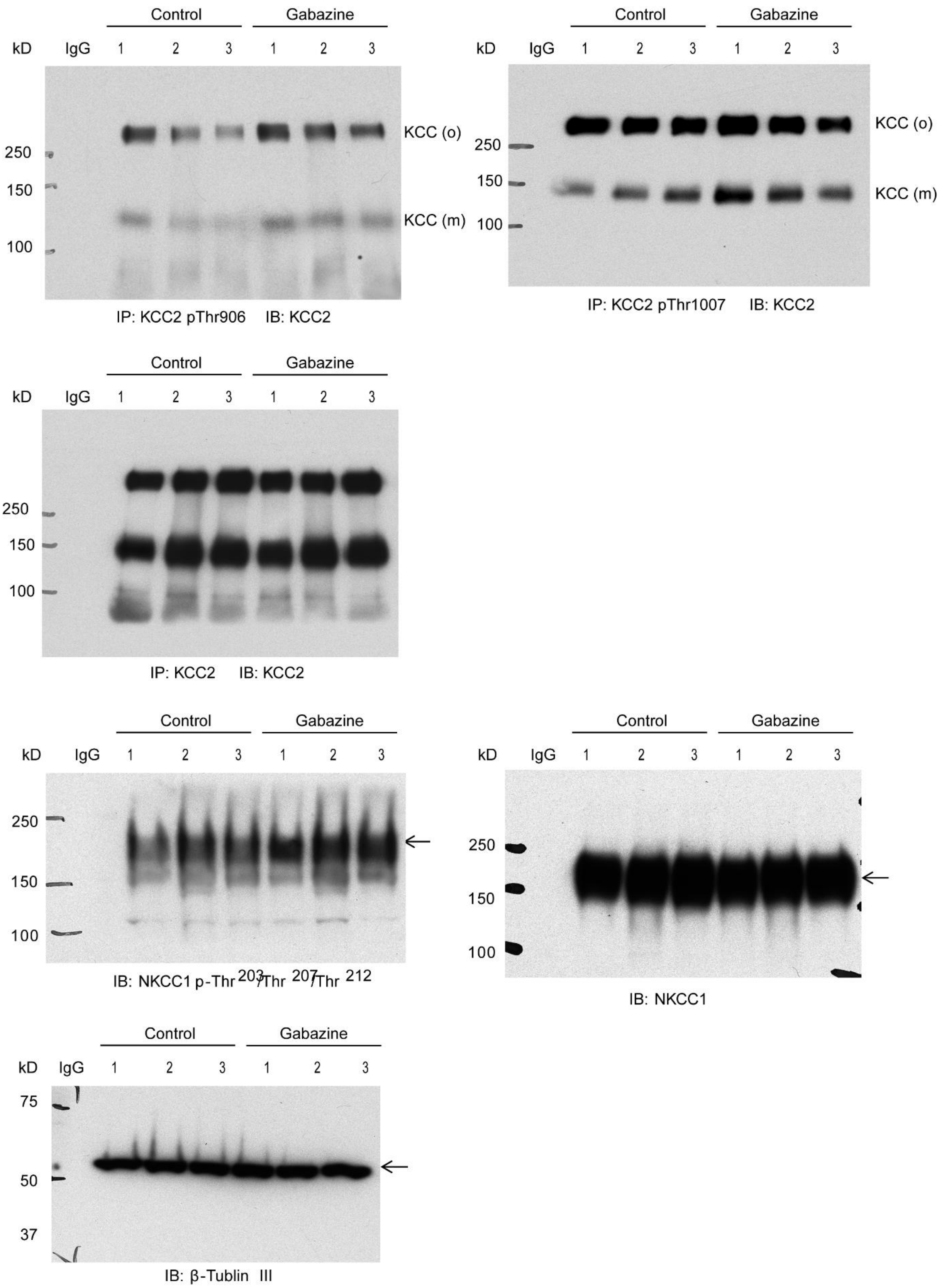

Supplementary Figure 7. Full size immunoblots related to Fig. 6A. *Molecular weight markers positions (kD).*

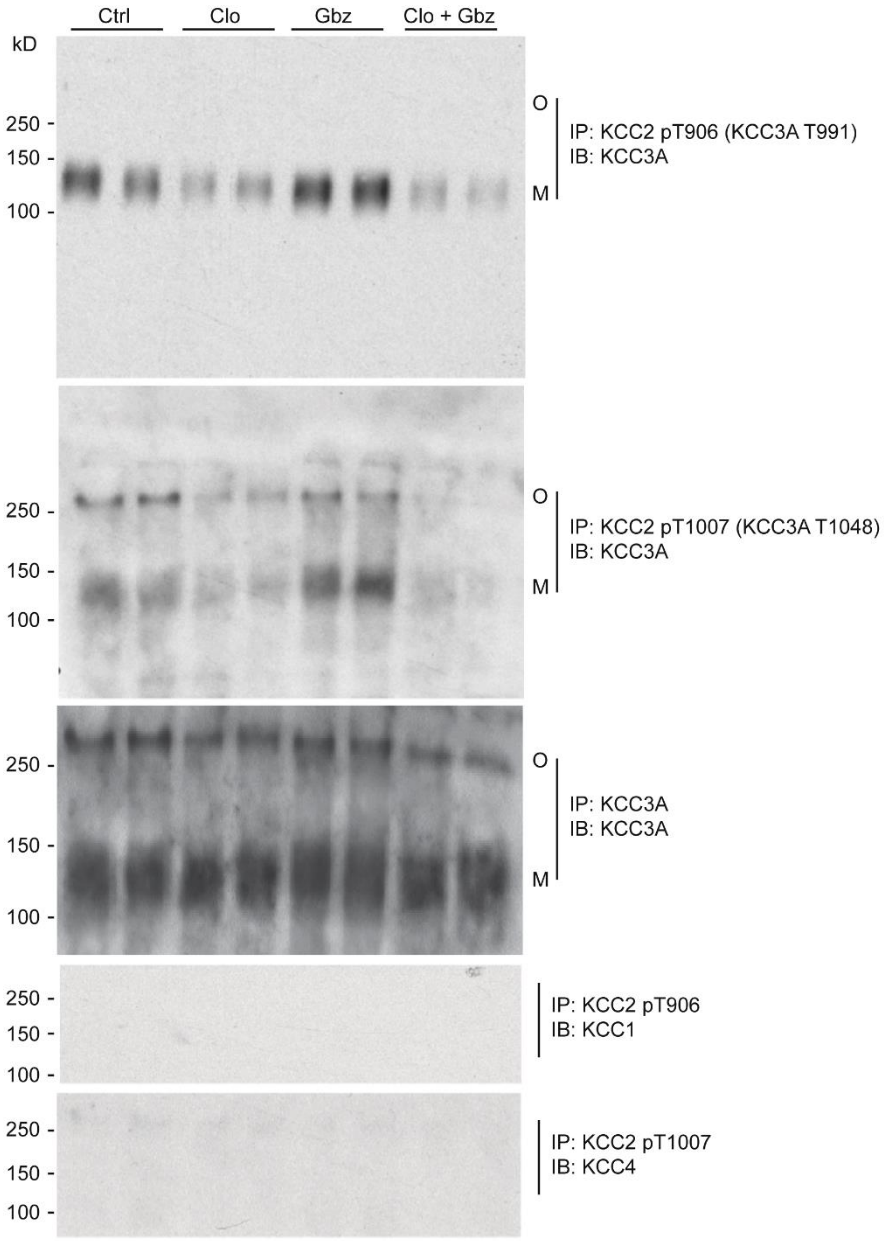

Supplementary figure 8. GABA$_A$R-dependent regulation of KCC3a threonine phosphorylation but not KCC1 or KCC4. *Western Blot of two independent experiments (1-2, 21DIV hippocampal cultures) of KCC3aT991/T1048 phosphorylation in gabazine (Gbz), closantel (Clo), or closantel+gabazine (clo+Gbz) conditions. Note IP with anti KCC2 pT906/1007 are recognized by an anti KCC3 antibody but not anti- KCC1 or KCC4 antibodies. Gabazine application increased phosphorylation of KCC3 on phospho-sites T991 and T1048 that is blocked by closantel.*

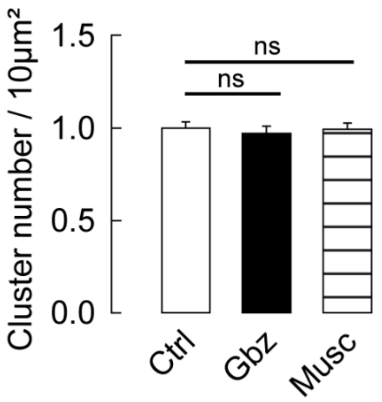

Supplementary figure 9. No effect of GABA$_A$R-mediated inhibition on KCC2 cluster density. *Flag surface staining in hippocampal neurons (DIV 23) expressing recombinant KCC2–Flag in absence (Ctrl) or presence of gabazine (Gbz) or muscimol (Musc) for 30 min. Quantifications showing no effect of gabazine (black, MW test p=0.539) or muscimol (pattern, MW test p=0.856) on KCC2 cluster number per dendritic unit length. Values were normalized to the corresponding control values. Ctrl n= 62 cells; Gbz n= 59 cells; Musc n= 58 cells; 4 cultures.*

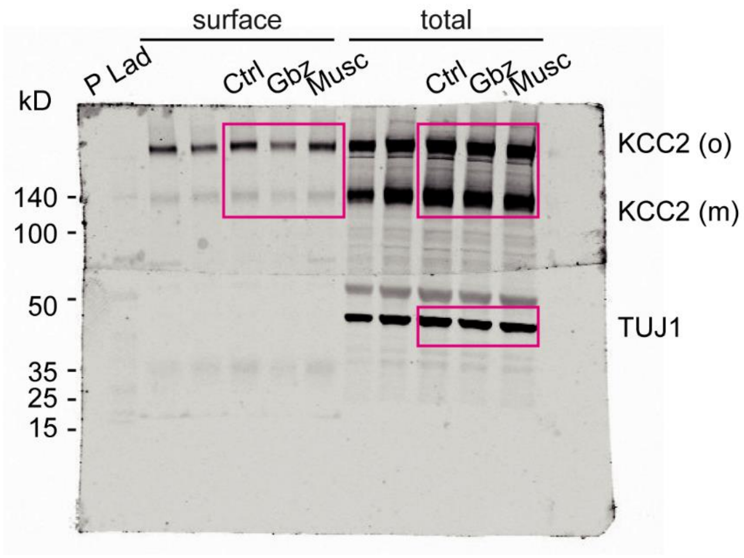

Supplementary Figure 10. Full size immunoblots related to Fig. 7C. *Red boxes indicate regions shown in the corresponding Figure. Molecular weight markers positions (kD).*

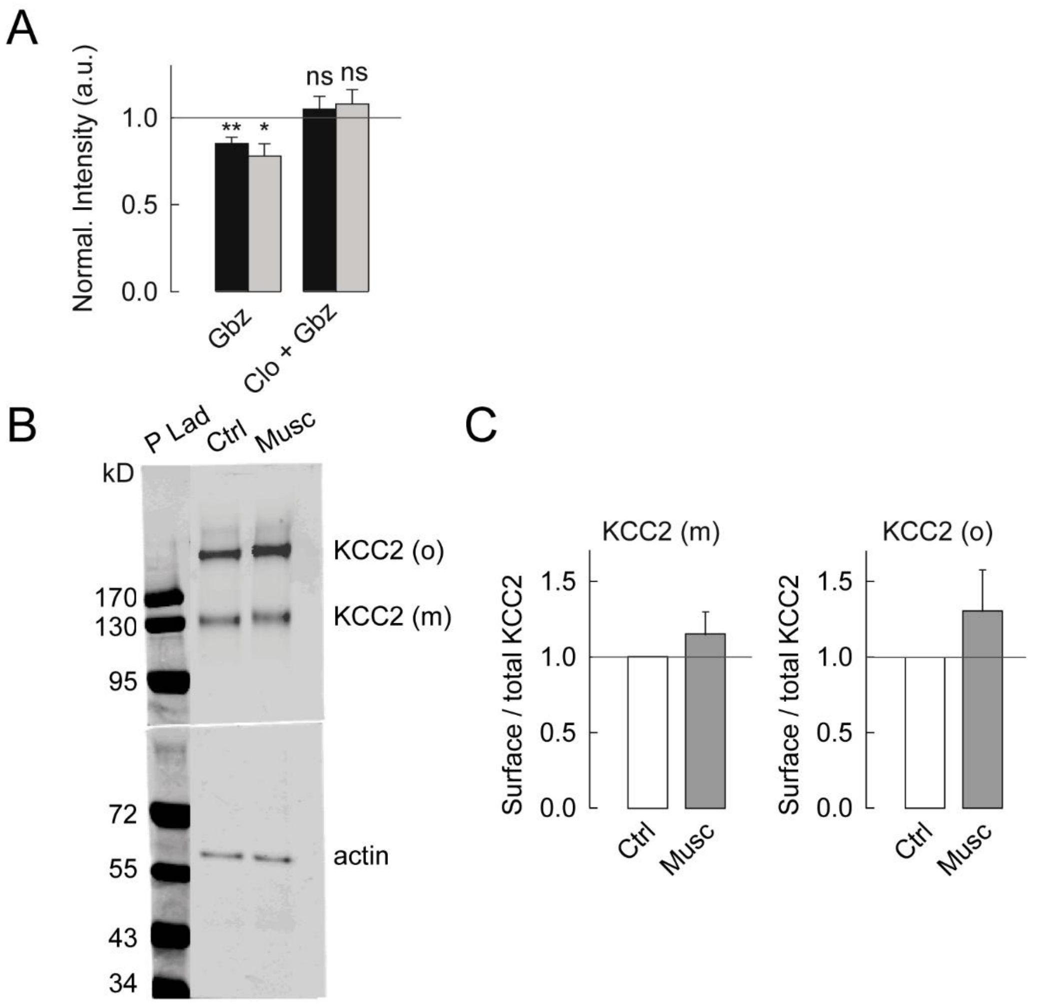

Supplementary figure 11. GABA$_A$R-dependent regulation of KCC2 in intact hippocampal network. A, *GABA$_A$R-mediated regulation of KCC2 in absence of Na+ channel blocker and glutamate receptors antagonists. Quantifications of KCC2 pixel (black) and cluster (gray) intensity in response to an acute application of gabazine or gabazine + closantel in absence of ttx+kynurenate+mCPG. Note the reduced transporter clustering after gabazine treatment and blockade of gabazine effect by closantel. Ctrl n= 64 cells, Gbz n= 65 cells, Closantel n= 50 cells, Closantel + gabazine n= 43 cells, 4 cultures, ctrl vs Gbz MW test p=0.004 and p=0.02 for pixel and cluster intensity respectively; Clo+Gbz vs Clo MW test p=0.686 and p=0.164 for pixel and cluster intensity.* ***B-C,*** *Acute GABA$_A$R activation increases the membrane stability of KCC2 in acute hippocampal slices.* ***B****, Biotinylation experiments showing an increase in the surface expression of KCC2 monomers (m) and oligomers (o) in 5-7 week old acute hippocampal slices after 30 min of muscimol treatment in absence of sodium channel and glutamate receptor blockers.* ***C****, Quantification of the ratio of the surface pool of KCC2 oligomers and monomers over the total pool of KCC2 in control (white) and muscimol (gray) conditions showing increase of surface KCC2 o and m after muscimol treatment. Values normalized to actin present in the non-biotinylated fraction. N=4 experiments. 3-5 slices per condition and per experiment. Rank sum test, KCC2m p=0.1 and KCC2o p=0.4.*

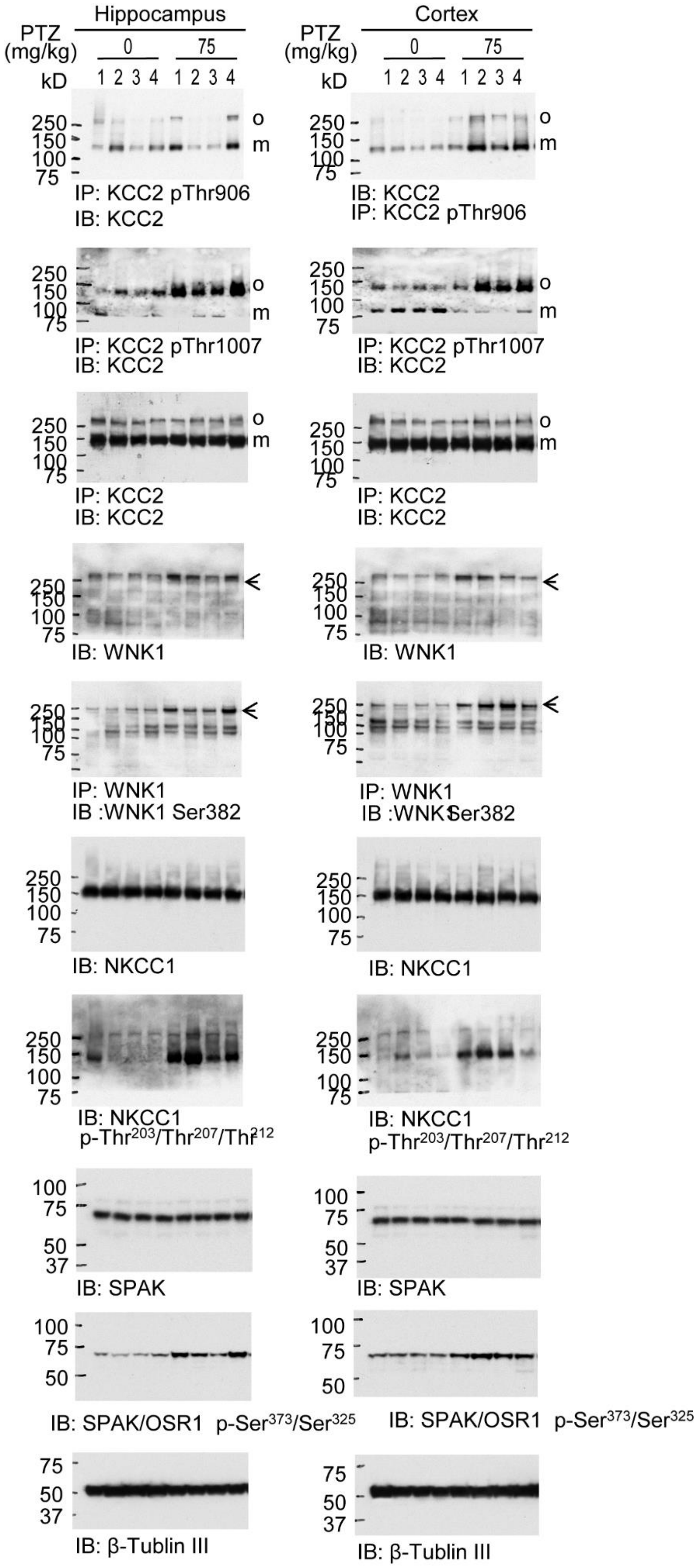

Supplementary Figure 12. Full size immunoblots related to Fig. 10A. *Molecular weight markers positions (kD).*

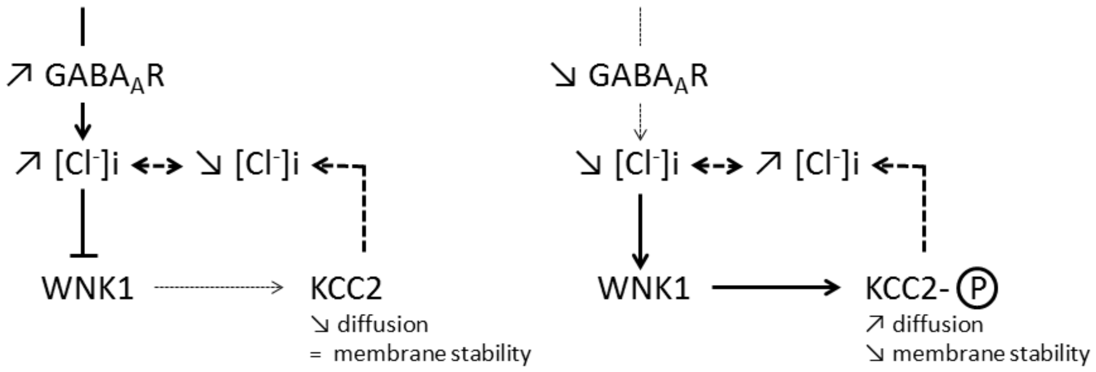

Supplementary Figure 13. Homeostatic control of intracellular chloride concentration via WNK1-dependent phosphorylation of KCC2. *Upon GABAAR activation, WNK1 gets inhibited by intracellular chloride leading to stabilization of KCC2 in the membrane and readjustment of [Cl-]i. In conditions of reduced GABAAR activity, lowering of [Cl-]i activates WNK1 and leads to removal of KCC2 from the membrane and elevation of [Cl-]i.*

**Experimental conditions**	**Fig. Nb**	**Median D (10^{-2} µm²s^{-1})**	**Fig. Nb**	**Median EA (10^{-3} µm²)**	**Fig. Nb**	**Mean DT (s)**
Bulk Ctrl	1B	2.7 (416, 38, 4)	1D	11.1 (838, 38, 4)	n.a.	n.a.
Bulk Muscimol	1B	2.6 (416, 33, 4)	1D	10.2 (838, 33, 4)	n.a.	n.a.
Bulk Ctrl	1F	2.4 (441, 45, 5)	1H	10.9 (880, 45, 5)	n.a.	n.a.
Bulk Gbz	1F	3,2 (441, 39, 5)	1H	14.8 (880, 39, 5)	n.a.	n.a.
Bulk Ctrl	S2A	3.6 (227, 15, 2)	S2B	15.9 (554, 15, 2)	n.a.	n.a.
Bulk Picrotoxin	S2A	4.4 (227, 12, 2)	S2B	18.3 (554, 12, 2)	n.a.	n.a.
Extra Ctrl	1J	2.1 (129, 25, 4)	1K	10.5 (362, 25, 4)	n.a.	n.a.
ES Ctrl	1J	1.9 (109, 33, 4)	1K	8.6 (212, 33, 4)	1L	11,9 (307, 33, 4)
IS Ctrl	1J	2,0 (89, 27, 4)	1K	8.6 (202, 27, 4)	1L	10,1 (162, 27, 4)
Extra Gbz	1J	3,0 (129, 25, 4)	1K	15.2 (362, 25, 4)	n.a.	n.a.
ES Gbz	1J	2,4 (109, 33, 4)	1K	10.9 (212, 33, 4)	1L	8,1 (218, 28, 4)
IS Gbz	1J	2,8 (89, 27, 4)	1K	11.0 (202, 27, 4)	1L	6,8 (119, 29, 4)

Supplementary table 1. GABA$_A$R-dependent control of KCC2 diffusion at synapses and at extrasynaptic sites. *Numbers in parentheses indicate the numbers of QDs, cells, and cultures analyzed. ES, QDs at excitatory synapses; IS, QDs at inhibitory synapses; Extra, QDs at extrasynaptic sites; n.a., not applicable.*

**Experimental conditions**	**Fig. Nb**	**Median D (10^{-2} µm²s^{-1})**	**Fig. Nb**	**Median EA (10^{-3} µm²)**
Ctrl	2A	1.9 (320, 12, 2)	2B	8.9 (640, 12, 2)
L655,708	2A	2.1 (320, 12, 2)	2B	8.9 (640, 12, 2)
Ctrl	2C	2.4 (271, 24, 3)	2D	8.6 (542, 24, 3)
2 µM GABA	2C	2.1 (271, 19, 3)	2D	7.8 (542, 19, 3)
2 µM GABA + L655,708	2C	2.7 (271, 19, 3)	2D	10.8 (542, 19, 3)
Ctrl	2E	2.5 (278, 19, 3)	2F	10.7 (555, 19, 3)
Baclofen	2E	2.5 (278, 19, 3)	2F	10.7 (555, 19, 3)
Ctrl	2E	2.5 (279, 23, 3)	2F	11.8 (580, 23, 3)
CGP52432	2E	2.4 (279, 20, 3)	2F	10.5 (580, 20, 3)

Cd^{2+}	3E	2.2 (250, 18, 3)	3F	11.6 (500, 18, 3)
Cd^{2+}+Gbz	3E	3.4 (250, 15, 3)	3F	14.7 (500, 15, 3)
S940D	3G	2.2 (190, 16, 3)	3H	10.4 (380, 16, 3)
S940D + Gbz	3G	2.6 (190, 15, 3)	3H	12.8 (380, 15, 3)

Supplementary table 2. Regulation of KCC2 diffusion by tonic GABA$_A$R-mediated inhibition but not by metabotropic GABA$_B$R and calcium signaling pathway. *Numbers in parentheses indicate the numbers of QDs, cells, and cultures analyzed.*

Experimental conditions	Fig. Nb	Median D (10^{-2} µm²s^{-1})	Fig. Nb	Median EA (10^{-3} µm²)
Ctrl	4D	2.8 (368, 22, 3)	4F	13.4 (712, 22, 3)
VU0240551	4D	2.5 (368, 22, 3)	4F	12.7 (712, 22, 3)
eNpHR t 0	4H	5.2 (215, 15, 2)	4J	22.5 (469, 15, 2)
eNpHR t 10s	4H	2.8 (215, 15, 2)	4J	11.5 (469, 15, 2)
eNpHR t 60s	4H	2.1 (215, 15, 2)	4J	7.7 (469, 15, 2)
138mM $[Cl^-]_{ex}$	4L	1.4 (408, 25, 3)	4N	5.6 (816, 25, 3)
0mM $[Cl^-]_{ex}$	4L	1.7 (408, 25, 3)	4N	7.3 (816, 25, 3)
Ctrl	5F	1.6 (405, 24, 4)	n.s.	7.5 (810, 24, 2)
Gbz	5F	2.2 (405, 20, 4)	n.s.	8.9 (810, 20, 2)
Closantel	5F	1.7 (405, 24, 4)	n.s.	8.2 (810, 24, 2)
Closantel + Gbz	5F	1.7 (405, 25, 4)	n.s.	5.7 (810, 25, 2)
shMock	5G,H	3.2 (322, 45, 5)	n.s.	15.8 (644, 45, 5)
shMock + Gbz	5H	3.9 (322, 31, 5)	n.s.	16.7 (644, 31, 5)
shWNK1	5H	3.7 (322, 41, 5)	n.s.	17.3 (644, 41, 5)
shWNK1 + Gbz	5H	3.5 (322, 31, 5)	n.s.	16.1 (644, 31, 5)
WNK1-KD	5G,H	3.5 (322, 34, 5)	n.s.	15.9 (644, 34, 5)
WNK1-KD + Gbz	5H	3.7 (322, 34, 5)	n.s.	15.4 (644, 34, 5)
WNK1-CA	5G	3.8 (322, 33, 5)	n.s.	17.0 (644, 33, 5)
shNT	S5E	1.0 (322, 18, 2)	n.s.	3.3 (644, 18, 2)
shNT + Gbz	S5E	2.1 (322, 16, 2)	n.s.	7.6 (644, 16, 2)
shWNK3	S5E	1.4 (330, 13, 2)	n.s.	5.8 (660, 13, 2)
shWNK3 + Gbz	S5E	2.1 (330, 31, 2)	n.s.	9.8 (660, 31, 2)
T906/T1007	6E,F	2.5 (197, 22, 3)	n.s.	10.9 (394, 22, 3)
T906/T1007 + Gbz	6F	3.4 (197, 15, 3)	n.s.	15.3 (394, 15, 3)
T906/T1007A	6E,F	2.7 (238, 19, 3)	n.s.	11.7 (476, 19, 3)
T906/T1007A + Gbz	6F	2.7 (238, 16, 3)	n.s.	10.7 (476, 16, 3)
T906/T1007E	6E,F	3.3 (241, 23, 3)	n.s.	16.1 (482, 23, 3)
T906/T1007E + Gbz	6F	3.3 (241, 15, 3)	n.s.	15.1 (482, 15, 3)

Supplementary table 3. Molecular mechanisms underlying GABA$_A$R-dependent regulation of KCC2 lateral membrane diffusion. *Numbers in parentheses indicate the numbers of QDs, cells, and cultures analyzed. n.s., not shown.*
